# Induction of heme oxygenase-1 by cobalt protoporphyrin enhances the antitumour effect of bortezomib in adult T-cell leukaemia cells

**DOI:** 10.1038/sj.bjc.6604003

**Published:** 2007-09-25

**Authors:** R S Hamamura, J H Ohyashiki, R Kurashina, C Kobayashi, Y Zhang, T Takaku, K Ohyashiki

**Affiliations:** 1 Department of Internal Medicine, Tokyo Medical University, Tokyo, Japan; 2 Intractable Immune System Disease Research Center, Tokyo Medical University, Tokyo, Japan; 3 Department of Clinical Pharmacology, Tokyo University of Pharmacy and Life Sciences, Tokyo, Japan

**Keywords:** adult T-cell leukaemia, proteasome inhibitor, bortezomib, heme oxygenase-1

## Abstract

Adult T-cell leukaemia (ATL) is a lethal neoplasia derived from HTLV-1-infected T lymphocytes frequently exhibiting nuclear factor-*κ*B (NF-*κ*B) activation. Despite the use of various treatment regimens, the prognosis of ATL is poor, and new treatment strategies are urgently needed. We therefore explored the effect and the molecular mechanism of a proteasome inhibitor, bortezomib, in ATL cells. We found bortezomib-induced cell death, and bortezomib suppressed constitutive NF-*κ*B activation via I-*κ*B stabilisation in three ATL cell lines (TaY, MT-2 and MT-4). An oligonucleotide DNA microarray analysis of TaY cells revealed upregulation of genes encoding heat shock proteins (HSPA1A, STIP1, HSPA1B, and HSPCA), genes related to protein folding (CDC37 and ANAPC5), Fas-associated factor 1(FAF1) and an oxidative stress-related gene, heme oxygenase-1(HMOX-1), known to be a target gene of hypoxia-inducible gene-1 alpha (HIF-1 alpha). Cobalt protoporphyrin induced HMOX-1, instead of HIF-1 alpha expression and increased bortezomib-induced apoptosis in the presence of pharmacologically effective doses of bortezomib. In contrast, zinc protoporphyrin downregulated HMOX-1 expression, thereby partially inhibiting bortezomib-induced cell death. This indicates that HMOX-1 may modulate anticancer effects of bortezomib in ATL cells, and could be a molecular target in treating ATL patients.

Adult T-cell leukaemia/lymphoma (ATL), an aggressive malignancy of CD4+ lymphocytes, is caused by the retrovirus HTLV-1 (Matsuoka and Jeang, 2005). Adult T-cell leukaemia/lymphoma is resistant to chemotherapy and has a very poor prognosis, which underlines the need for new therapeutic approaches. Leukaemia is preceded by oligoclonal expansion of HTLV-1-infected activated T cells, as a result of the viral transactivator protein Tax expression, which activated various cellular genes including CREB/ATF, AP1, and NF-*κ*B (Sun and Yamaoka, 2005).

Activated nuclear factor-kappa B (NF-*κ*B) was reported in HTLV-1-associated cells, and has been implicated in resistance to anticancer agents and apoptosis. In resting lymphocytes, NF-*κ*B is sequestrated in cytoplasm, where it is inactivated by association with inhibitory I-*κ*B subunit (Pierce *et al*, 1997). Upon stimulation, I-*κ*B*α* protein is phosphorylated, and subsequently degraded by the proteasome, resulting in the release of an active NF-*κ*B that translocates into the nucleus and subsequently activates the transcription of the target genes (Karin and Ben-Neriah, 2000). Tax can activate the NF-*κ*B pathways through direct interaction with the IKK complex, which causes I-*κ*B*α* to be phosphorylated, ubiquitylated, and subsequently degraded by proteasomes; however, *de novo* ATL cells frequently lose Tax expression, thereby escaping from cytotoxic T lymphocytes (Furukawa *et al*, 2006).

The proteasome inhibitor, bortezomib (also known as VELCADE or PS-341), represents a new class of anticancer drugs which has been shown to inhibit the growth and/or progression of human cancers, including multiple myeloma (Mitsiades *et al*, 2002; Rajkumar *et al*, 2005; Cavo, 2006; Fisher *et al*, 2006). Although it has been theoretically hypothesised that bortezomib abrogates the degradation of I-*κ*B, which blocks the transcriptional activity of NF-*κ*B, recent studies demonstrated that bortezomib elicits activation of multiple pathways in cancer cells, such as endoplasmic reticulum (ER) stress and reactive oxygen species (ROS) pathways (Fribley *et al*, 2004; Nawrocki *et al*, 2005; Obeng *et al*, 2006; Pérez-Galán *et al*, 2006; Yang *et al*, 2006). More recently, HIF-1 alpha has been shown to determines the sensitivity of endothelial cells to the proteosome inhibitor bortezomib (Veschini *et al*, 2007).

Despite the fact that bortezomib affects several pathways critical for the survival of HTLV-1-positive and -negative malignant T cells (Satou *et al*, 2004; Nasr *et al*, 2005), the underlying mechanism by which bortezomib inhibits ATL cell growth has not been fully elucidated. We therefore attempted to clarify the molecular pathway which might be involved in bortezomib-induced cell death.

## MATERIALS AND METHODS

### Reagents and cell cultures

Bortezomib was kindly provided by Millennium Pharmaceuticals Inc. (Cambridge, MA, USA). In all experiments, an IL-2-dependent ATL cell line, TaY, which has been established and well characterised in our laboratory (Zhou *et al*, 1998; Ojima *et al*, 2005; Takaku *et al*, 2005), was used in this study. Early passages of TaY cells were retrieved and grown in RPMI 1640 with 10% FBS and 20 U ml^−1^ of IL-2 (Roche Diagnostics, Mannheim, Germany). Two IL-2-independent ATL cell lines (MT-2 and MT-4) (Miyoshi *et al*, 1982), and an HTLV-1-negative T-cell line (Jurkat) (Gillis and Watson, 1980) were also used in a part of our study. After obtaining written informed consent, peripheral blood mononuclear cells (PBMCs) were isolated from two patients with ATL by Ficoll-Hypaque. Cobalt protoporphyrin (CoPP) was purchased from Frontier Scientific (Logan, UT, USA). Zinc protoporphyrin (ZnPP) was purchased from Alexis Biochemicals (San Diego, CA, USA).

### Cell viability and apoptosis assay

The inhibitory effect of bortezomib on cell growth was assessed by a cell counting kit (Wako Chemicals, Tokyo, Japan). Briefly, the cells (5000 well^−1^) were incubated in triplicate in a 96-well plate in the presence or absence of indicated test samples at a final volume of 0.1 ml for 24 h at 37°C. Thereafter, 0.01 ml of tetrazolium salt, WST-1 was added to each well. After 2-h incubation at 37°C, the optical density (OD) at 450 nm was measured using a 96-well multiscanner autoreader with the extraction buffer used as a blank. Cell viability was expressed as a percentage (OD of the experiment sample/OD of the control × 100). For detection of apoptosis, the Annexin V-binding capacities of the treated cells were examined by the Annexin V-biotin apoptosis detection kit (Calbiochem, La Jolla, CA, USA) using an Agilent 2100 Bioanalyzer (Agilent, Wilmington, DE, USA), according to the supplier’s instructions.

### Evaluation of NF-*κ*B activity

The DNA-binding activity of NF-*κ*B was quantified by enzyme-linked immunosorbent assay (ELISA) using Trans-AM NF-*κ*B p65 Transcription factor assay kit (Active Motif North America, Carlsbad, CA, USA).

### Western blotting

Cells were washed twice in phosphate-buffered saline (PBS) and cell pellets were lysed in electrophoresis buffer and boiled for 10 min. The equivalent of 30 *μ*g protein was loaded onto each lane of a 10% tris-glycine gel. The separated protein was blotted onto a filter. After blockage of nonspecific binding sites with BlockAce (Dainippon-Sumitomo Pharma, Osaka, Japan), the filter was incubated with antibodies for 60 min at room temperature; anti-I*κ*B (Abcam Plc, Cambridge, UK), anti-HMOX-1 (Stressgen Bioreagents, Ann Arbor, MI, USA), anti-HIF (Novus Biologicals, Inc., Littleton, CO, USA) and anti-GAPDH (Abcam Plc). After washing, the blots were incubated for 60 min with horseradish peroxidase (HRP)-linked anti-mouse IgG (Amersham Biosciences, Buckinghamshire, UK). Signals were visualised using an ECL western blotting detection reagents and analysis system (Amersham Biosciences).

### RNA isolation and labelling

TaY cells were treated with 0.5–100 nM of bortezomib for 24 h. After treatment, cells were harvested, and total RNA was extracted using an RNeasy Mini Kit (Qiagen, Germantown, MD, USA). The amount of RNA was measured by NanoDrop (NanoDrop Technologies, Wilmington, DE, USA), then the quality of extracted RNA was checked using a 2100 Bioanalyzer (Agilent Technologies, Wilmington, DE, USA). A Label Star array kit (Qiagen) was used for cDNA labelling according to the supplier's instructions as reported previously (Zhang *et al*, 2006).

### Oliginucleotide DNA microarray

We designed a pathway-focused low-density oligonucleotide microarray (Novusgene Inc., Tokyo, Japan) which contains 667 selected genes related to cell growth, cell cycle, apoptosis, transcription, DNA repair and cell stress responses (GPL 3837). For dual-colour analysis, cDNA obtained from untreated TaY cells was labelled with Cy3 as a reference, and cDNA obtained from the bortezomib-treated cells was labelled with Cy5. Hybridisation was carried out automatically using GeneTAC Hybstation (Genomic Solutions, Ann Arbor, MI, USA), as reported previously (Takaku *et al*, 2005).

### Data analysis and statistic validation

The hybridisation signals were scanned by GenePix 4000B (Axon Instruments, Union City, CA, USA). For statistical analysis of gene expression, we utilised a GeneSifter® (VizXLabs, Seattle, WA, USA). Analysis of variance (ANOVA) and Student's *t*-test were done using GeneSifter®. *P*-values of less than 0.05 were considered to indicate a statistically significant difference and the Benjamini–Hochberg algorithm was used for estimation of false discovery rates (Zhang *et al*, 2006).

### Real-time reverse transcriptase–PCR (RT–PCR)

To confirm the microarray results, we performed RT–PCR by ABI Prism 7700 Sequence Detection System (Applied Biosystems, Foster City, CA, USA) as reported elsewhere (Ohyashiki *et al*, 2005). We utilised Taqman gene expression assays (Applied Biosystems), and the amount of gene expression in each sample was evaluated as a percent with respect to the standard curve generated from a serial dilution of quantitative PCR human reference total RNA (Stratagene, La Jolla, CA, USA). The obtained data from GAPDH were used to standardise the sample variation in the amount of input cDNA.

## RESULTS

### Bortezomib-induced cell death in ATL cell lines

**Figure 1:**
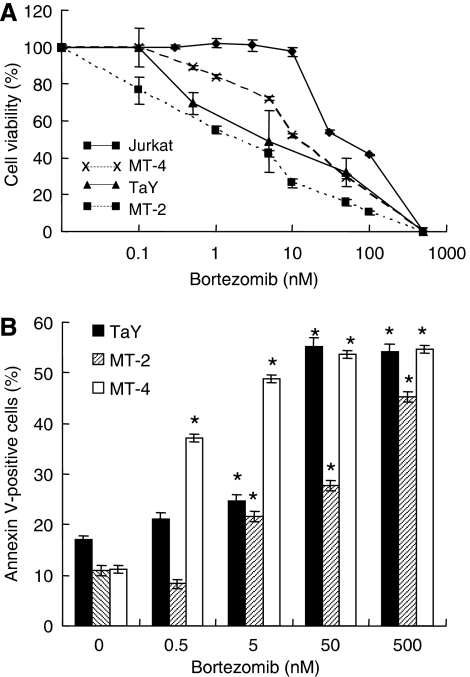
(**A**) Cytotoxity of bortezomib on TaY, MT-2, MT-4 and Jurkat. Cells were cultured in the presence or absence of increasing doses of bortezomib (1–500 nM). The percentage of viability is plotted with respect to untreated cells. The results are shown as means (±s.d.) percentage of viability from triplicate cultures with repeated experiments. (**B**) Bortezomib induced apoptosis in ATL cell lines (TaY, MT-2 and MT-4). Cells were cultured in the presence of bortezomib for 24 h. Cell death was analysed by an annexin V-biotin apoptosis detection kit. Apoptosis is expressed as a percent of Annexin V-positive cells in bortezomib-treated cells.

We first examined the effect of bortezomib on ATL cell lines, TaY, MT-2, MT-4 and a HTLV-1-negative T-cell line, Jurkat, *in vitro* by a cell-counting assay. The median inhibitory concentration (IC_50_) of bortezomib for TaY was 5 nM, that for MT-2 was 4.5 nM, that for MT-4 was 10 nM and that for Jurkat was 30 nM, respectively (Figure 1A). The expression level of Tax was not affected by bortezomib-treated ATL cells (data not shown). To study whether or not the cell death induced by bortezomib was apoptosis, we analysed the bortezomib-induced cell death by annexin V staining. Cells were treated for 24 h with different concentrations of bortezomib, then stained with annexin V-Cy5. The results showed apoptosis in a dose-dependent manner, indicating that bortezomib induced apoptosis of ATL cell lines (Figure 1B); cell death was also confirmed by morphology (Supplementary file).

### Bortezomib suppresses constitutive NF-*κ*B activation via I-*κ*B stabilisation in ATL cell lines

**Figure 2:**
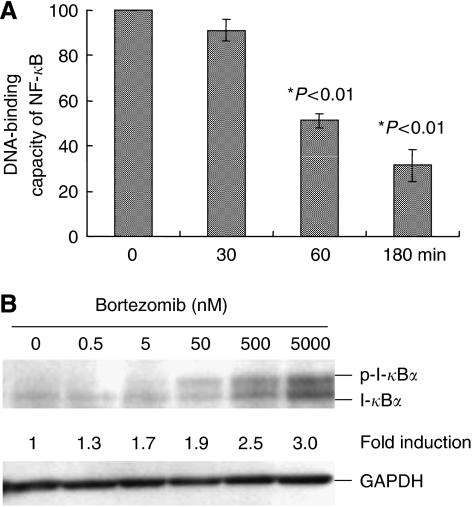
(**A**) DNA-binding capacity of NF-*κ*B in TaY cells. TaY cells were cultured in the presence of bortezomib for the indicated periods (0–180 min), harvested and subjected to EMSA. (**B**) Expression of I-*κ*B*α* in TaY cells. Whole cell extracts (30 *μ*g/lane) of TaY cells are immunoblotted with specific antibodies against I-*κ*B*α* and GAPDH. Accumulation of I-*κ*B*α* and phosphorylated I-*κ*B*α* were observed in a dose-dependent manner.

We then investigated the DNA binding of NF-*κ*B by an ELISA assay. Cells were treated with bortezomib for different periods of time. We found the DNA-binding capacity of NF-*κ*B decreased in a time-dependent manner, indicating an inhibitory effect of 5 nM of bortezomib on NF-*κ*B activation in TaY cells (Figure 2A). The decreased activity of NF-*κ*B was also evident in MT-2 and MT-4 after 3 h exposure to bortezomib; DNA-binding activity of NF-*κ*B after bortezomib treatment was 26.8% in MT-2 and 17.0% in MT-4 cells, respectively, compared to those in untreated cells. In order to clarify the inhibitory effect of bortezomib on the NF-*κ*B/I-*κ*B cascade, we analysed the protein level of I-*κ*B*α* in TaY cells after 24 h treatment with bortezomib. Consistent with the decreased DNA-binding activity of NF-*κ*B, bortezomib induced the accumulation of I-*κ*B*α* in TaY cells (Figure 2B). Accumulation of I-*κ*B*α* was also confirmed in MT-2 and MT-4.

### Gene expression profile of bortezomib-treated TaY cells

**Figure 3:**
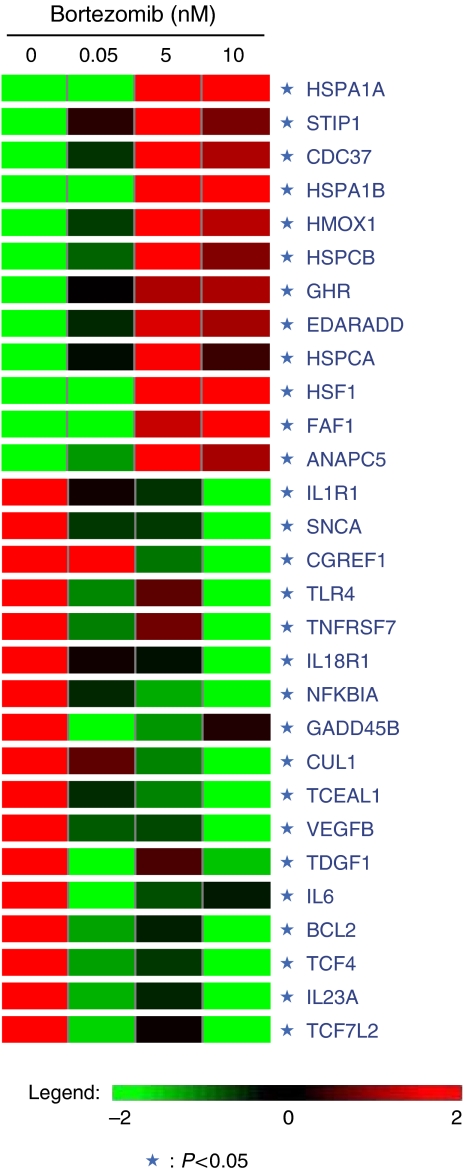
The molecular signature of bortezomib-treated TaY cells. ANOVA analysis was performed by GeneSifter® using microarray analysis data deposited in GEO (GSE5794). Genes whose expression levels are greater (red) or lower (green) in bortezomib-treated TaY cells compared to those in untreated cells were navigated by GeneSifter®. They are also listed in Table 1.

**Figure 4:**
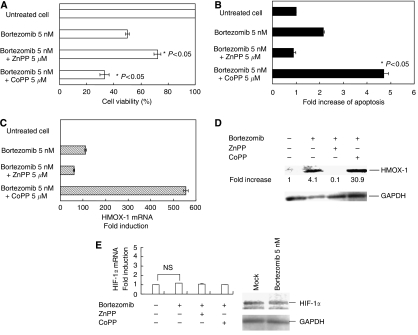
Effect of CoPP and ZnPP in bortezomib-induced cell death. TaY cells were cultured with CoPP or ZnPP in the presence of bortezomib. (**A**) The percentage of cell viability was plotted with respect to untreated cells. (**B**) Apoptosis was expressed as fold increases of annexin V-positive cells in bortezomib-treated cells compared to those in untreated cells. (**C**) Fold induction (or reduction) of HMOX gene expression measured by real-time RT–PCR. (**D**) Western blotting of HMOX-1 and GAPDH. Fold increases were expressed as a signal of HMOX-1/GAPDH in the specimen with respect to those in untreated cells. (**E**) HIF-1 alpha expression in bortezomib-treated TaY cells (real-time RT–PCR, left; western blotting, right).

To further understand how bortezomib induced apoptosis in ATL cells, we used TaY cells treated with bortezomib or vehicle control for short time periods, and microarray analysis was performed using in-house prepared oligonucleotide DNA microarrays (NCBI Gene expression omnibus, GSE5794, unreleased). Differential expression was analysed using a GeneSifter® (Figure 3). Upregulated or downregulated genes in bortezomib-treated TaY cells (expression level in the sample was four-fold greater or lower than in untreated TaY cells) are summarised in Table 1. In TaY cells, bortezomib induced upregulation of genes encoding heat shock proteins (HSPA1A, STIP1, HSPA1B and HSPCA), genes related to protein folding (CDC37 and ANAPC5) and Fas-associated factor 1(FAF1) known as an apoptosis facilitator. Upregulation of HMOX-1, which is known to be a target gene of hypoxia-inducible gene-1 alpha (HIF-1 alpha), was notable in bortezomib-treated TaY cells. The results obtained from microarray data were consistent with those analysed by real-time RT–PCR and the induction of HMOX-1 after treatment with pharmacological concentrations of bortezomib was more than 100-fold compared to untreated cells (Figure 4C). Downregulation of vascular endothelial growth factor (VEGF) beta, IL6 and BCL2 were also evident, as previously identified in human multiple myeloma cells following bortezomib-induced apoptosis. Downregulation of a subset of cytokines and cytokine receptors (i.e. IL1R1, IL18R1, IL23A) was also noted.

### Induction of HMOX-1 by CoPP enhances the antitumour effect of bortezomib in ATL cells

Based on the results obtained from the differential expression pattern, we in particular focused our study on a gene closely related to oxygen stress, i.e., HMOX-1. To determine the possible role of HMOX-1 in bortezomib-induced apoptosis in TaY cells, we examined the change of apoptotic effect by adding the HMOX-1 inducer, CoPP, or the HMOX-1 inhibitor, ZnPP. Although CoPP and ZnPP did not affect the cell growth of TaY cells in the absence of bortezomib (data not shown), we found that CoPP significantly enhanced the cytotoxity and bortezomib-induced apoptosis in TaY cells (Figure 4A and B). In contrast, ZnPP partially inhibited bortezomib-induced cell death in TaY cells with pharmacologically effective dose of bortezomib (Figure 4A and B). Real-time RT–PCR and western blot analysis revealed that CoPP significantly enhanced HMOX-1 in TaY cells (Figure 4C and D). To determine whether HMOX-1 induced the antiproliferative activity of bortezomib via HIF-1 alpha or not, we performed real-time RT–PCR; however, upregulation of HIF-1 alpha was not evident (Figure 4E left). Western blot analysis also revealed that HIF-1 alpha was not significantly elevated after treatment of bortezomib in TaY cells (Figure 4E right). This indicates that upregulation of HMOX-1 rather than HIF-1 alpha affects the antitumour effect of bortezomib.

**Figure 5:**
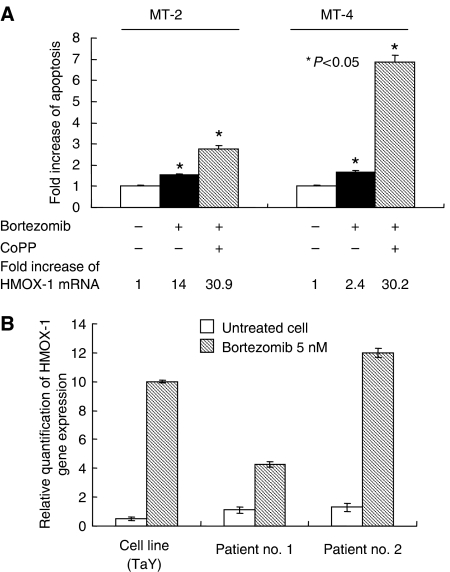
(**A**) Induction of HMOX-1 by CoPP enhances apoptotic effects of bortezomib in MT-2 and MT-4 cells. Fold increase of HMOX-1 mRNA is shown at the bottom of the graph. (**B**) Comparison of gene expression levels between cell line (TaY) and patient sample. Percent of ATL cells in the blood specimens is 46% in patient no.1 and 82% in patient no. 2, respectively. Relative gene expression levels are expressed as ratios (copy numbers of target gene/copy numbers of GAPDH). In the presence of bortezomib, upregulation of HMOX-1 is evident in both TaY cells and patient samples.

To ascertain whether involvement of HMOX-1 in bortezomib-induced cell death is not restricted in TaY cells, we analysed the effect of CoPP in MT-2 and MT-4. Upregulation of HMOX-1 by CoPP enhanced sensitivity to bortezomib in MT-2 and MT-4, indicating that this phenomenon is consistent with the results obtained from TaY cells (Figure 5A).

### Gene expression levels of HMOX-1 in bortezomib-treated ATL patient’ sample

To validate whether genes extracted in the current study indeed reflect molecular pathways in bortezomib-treated ATL cells, we analysed the gene expression levels of HMOX-1 in bortezomib-treated TaY cells and ATL cells obtained from two patients. The HMOX-1 expression level was remarkably elevated in bortezomib-treated fresh ATL cells as well as an ATL cell line (TaY). This indicates that the molecular pathway involving HMOX-1 may play an important role in *de novo* ATL cells treated with bortezomib (Figure 5B).

## DISCUSSION

In this study, we demonstrated that the mechanism of the antitumour effect of bortezomib on ATL cells involves oxygen-dependent gene regulation in addition to the I-*κ*B*α* /NF-*κ*B pathway. Based on the results obtained from gene expression profiling, we in particular focused on HMOX-1, as representative of oxygen-dependent gene regulation in bortezomib-induced apoptosis. We found that bortezomib induced HMOX-1 expression at the transcriptional level as well as at the protein level. HMOX-1, an inducible enzyme that catalyses oxidative degradation of heme to form biliverdin, carbon monoxide and free iron has recently been implicated in the regulation of angiogenesis via VEGF activity (Morse and Choi, 2005). Evidence suggests that expression of HMOX-1 is induced in response to ROS (Morse and Choi, 2005). Bortezomib was reported to induce ROS generation in various cancer cells such as non-small-cell lung cancer, leukaemia cells, and head and neck squamous cell carcinoma. ROS was therefore implicated as the primary cause for bortezomib-induced apoptosis of those cells (Ling *et al*, 2003; Fribley *et al*, 2004; Yu *et al*, 2004; Landowski *et al*, 2005). Recent reports have demonstrated that NF-*κ*B indirectly inhibits ROS generation (Sakon *et al*, 2003; Kamata *et al*, 2005); however, the intracellular ROS balance may be regulated by multiple pathways, therefore, it is still uncertain whether ROS generation is due to NF-*κ*B/I-*κ*B inhibition cascade in bortezomib-treated cancer cells.

Oxygen regulation is a complex process that has special relevance in the progression of cancer. Cells subjected to hypoxia (reduced ability of oxygen) undergo transcriptional changes that promote increased anaerobic metabolism, angiogenesis and other adaptive responses (Semenza, 2003). The key mediator of these changes is the transcription factor HIF-1, a heterodimer of the protein subunits HIF-1 alpha, which is induced by hypoxia and HIF-1 beta, which is constitutively expressed (Wang *et al*, 1995). HIF-1 alpha protein is mainly regulated post-transcriptionally by inhibiting its ubiquitination and proteolysis (Huang *et al*, 1998). It is known that HIF-1 alpha ultimately leads to HIF-1-regulated gene expression, such as HMOX-1 and VEGF (Bussolati and Mason (2006)). We therefore assumed that bortezomib induced upregulation of HIF-1 alpha by inhibiting proteolysis, thereby, upregulation of HMOX-1 via HIF-1 alpha leads to bortezomib-induced cell death. In the current study, however, upregulation of HIF-1 alpha was not evident at either the transcriptional level or the protein level in TaY cells.

Cobalt protoporphyrin is known to be a potent and effective inducer of HMOX-1 in many tissues. Unlike haem, CoPP is neither a pro-oxidant nor a substrate for HMOX-1, therefore, it might be considered as a potential therapeutic agent in situations where upregulation of HMOX-1 is desired. In the current study, CoPP induced HMOX-1 expression. Instead, HIF-1 alpha expression was downregulated by CoPP, possibly due to a feedback mechanism for upregulation of HMOX-1. This further suggests that bortezomib modulates the oxygen-dependent gene regulation, and induction of oxygen-dependent genes may serve as a feedback mechanism to balance the intracellular level of ROS. Since HIF-1 alpha activates the transcription of genes that are involved in cell survival as well as apoptosis, we could not completely rule out the possibility that subtle change of HIF-1 alpha modulates bortezomib-induced cell death via pathways other than those involving HMOX-1. To the best of our knowledge, this is the first report that induction of HMOX-1 by CoPP enhances bortezomib-induced apoptosis.

Although we were able to identify only a limited number of genes related to bortezomib-induced cell death in ATL cells, we demonstrated that HMOX-1 regulation plays an important role in the antitumour activity of bortezomib. Since the number of patients studied was too small to draw a definite conclusion, it should obviously be clarified in large numbers of patient specimen; however, the enhanced sensitivity of ATL cells to bortezomib by CoPP is intriguing and suggests that HMOX-1 may be an attractive target for treating ATL patients.

## Figures and Tables

**Table 1:** Identification of genes affected by bortezomib treatment

**Gene accession #**	**Gene name**		***P*-value (ANOVA)**
*(a) Upregulated genes whose expression level was greater than four-fold compared to those in untreated cells*			
NM_005345	HSPA1A	Heat shock 70 kDa protein 1A	0.000012
NM_006819	STIP1	Stress-induced-phosphoprotein 1 (Hsp70/Hsp90-organizing protein)	0.000037
NM_007065	CDC37	CDC37 cell division cycle 37 homologue	0.000036
NM_005346	HSPA1B	Heat shock 70 kDa protein 1B	0.000001
NM_002133	HMOX1	Heme oxygenase (decycling) 1	0.015313
NM_007355	HSPCB	Heat shock protein 90 kDa alpha (cytosolic), class B member 1 (HSP90AB1)	0.000033
NM_000163	GHR	Growth hormone receptor	0.001547
NM_080738	EDARADD	EDAR-associated death domain	0.000078
NM_005348	HSPCA	Heat shock protein 90 kDa alpha (cytosolic), class A member 1 (HSP90AA1), transcript variant 2	0.00178
NM_005526	HSF1	Heat shock transcription factor 1	0.006578
NM_007051	FAF1	Fas (TNFRSF6)-associated factor 1	0.006247
NM_016237	ANAPC5	Anaphase-promoting complex subunit 5	0.011988

*(b) Downregulated genes whose expression level was lower than four-fold compared to those in untreated cells*			
NM_000877	IL1R1	Interleukin 1 receptor, type I	0.012833
NM_000345	SNCA	Synuclein, alpha	0.000001
NM_006569	CGREF1	Cell growth regulator with EF-hand domain 1	0.004154
NM_138556	TLR4	Toll-like receptor 4	0.000122
NM_001242	TNFRSF7	Tumour necrosis factor receptor superfamily, member 7	0.000002
NM_003855	IL18R1	Interleukin 18 receptor 1	0.007924
NM_020529	NFKBIA	Nuclear factor of kappa light polypeptide gene enhancer in B-cells inhibitor, alpha	0.000013
NM_015675	GADD45B	Growth arrest and DNA-damage-inducible, beta	0.000001
NM_003592	CUL1	Cullin 1	0.008025
NM_004780	TCEAL1	Transcription elongation factor A (SII)-like 1	0.000001
NM_003377	VEGFB	Vascular endothelial growth factor B	0.000002
NM_003212	TDGF1	Teratocarcinoma-derived growth factor 1	0.000759
NM_000600	IL6	Interleukin 6	0.01754
NM_000633	BCL2	B-cell CLL/lymphoma 2 (BCL2)	0.000054
NM_003199	TCF4	Transcription factor 4	0.000052
NM_016584	IL23A	Interleukin 23, alpha subunit p19	0.000001
NM_030756	TCF7L2	Transcription factor 7-like 2	0.000006

List of genes in Table 1 correspond to the heat map shown in Figure 3. Gene accession number, full name of each gene and statistical significance are shown above.
