# Supplementary material for: Induction of heme oxygenase-1 by cobalt protoporphyrin enhances the antitumour effect of bortezomib in adult T-cell leukaemia cells
Source: Br J Cancer. 2007 Sep 25;97(8):1099–105. doi: 10.1038/sj.bjc.6604003 (PMC2360455; doi:10.1038/sj.bjc.6604003)
Supplement: Supplementary File [file 6604003x1.ppt]

## Slide 1
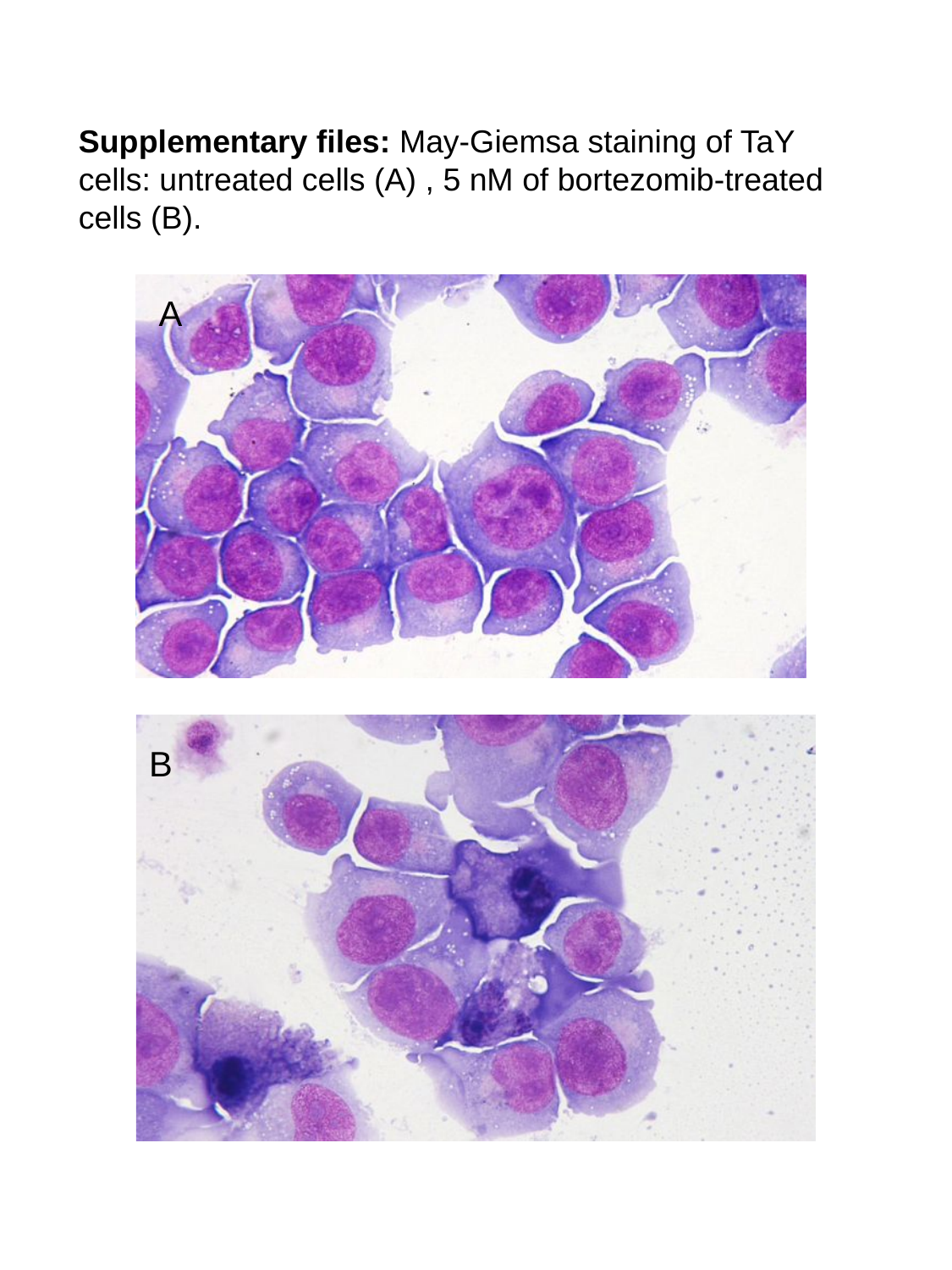

Supplementary files: May-Giemsa staining of TaY cells: untreated cells (A) , 5 nM of bortezomib-treated cells (B).
A
B
